# Almoners and Children's Aid Committees

**Published:** 1913-07-12

**Authors:** 


### A POINT IN ADMINISTRATIVE MEDICINE.

### Almoners and Children's Aid Committees.

A correspondent writes to us as follows, *à propos* of the references to the work of the Invalid Children's Aid Association in The Hospital, May 24, 1913, page 245:—

The sympathetic allusions to the work of the Invalid Children's Aid Association in your recent article on the provision of surgical appliances for hospital patients are well deserved, as I can personally testify. Naturally your contributor looks at the matter mainly from the almoner's point of view; and it is very gratifying to find that the work of the Invalid Children's Aid Association is so much appreciated by those who have to deal with these problems in the voluntary hospitals.

Speaking broadly, the hospital almoner and the local executive of the Invalid Children's Aid Association should be, and usually are, in close touch with one another. This closeness of contact, to be perfectly frank, occasionally breeds friction, a thing which is entirely regrettable and, as a rule, quite unnecessary. If any proof that differences of opinion do sometimes arise is needed it would be furnished by the course of a debate in the last week of June at the Central Council of the Invalid Children's Aid Association. Certain grievances, real or fancied, were ventilated concerning the difficulties which some branches have met with in their dealings with hospital almoners; and although, of course, one side of the question was much more prominent in the discussion than the other, it did appear as if some almoners, at least, occasionally show want of discrimination and tact in dealing with the Invalid Children's Aid Association. As it happened, both a resolution which was proposed, and an amendment to it, were lost when put to the vote, thus leaving matters *in statu quo*; and probably this unwillingness of the Central Council to attempt to interfere between the local branches and individual almoners by any rigid regulations is the best policy.

Satisfactory as this decision is, it is nevertheless noteworthy that several delegates from branches seemed to think a more vigorous policy advisable. It was alleged that in a few cases almoners have got into a habit of arranging admission to convalescent homes for hospital patients, and then sending the bill, as it were, to be footed by the Invalid Children's Aid Association. It is not strange that such usurpation of function produces resentment. Another complaint was that almoners in some cases supply such bald summaries of medical and other certificates that branches of the Invalid Children's Aid Association are quite unable to form any useful opinion what form of after-care and treatment will best suit the case. When, as is nearly always the case, the local branch has a doctor or two present at its committee meetings, technical details of the medical aspects of the case are not likely to be misinterpreted or misunderstood; and almoners would do well to assume that their communications will be discussed by committees thus assisted by experts, and that vague or non-committal medical certificates will come in for criticism.

Enough has been said to show that some irritation has been engendered. Quite possibly the almoners may be able to retort with a list of shortcomings of the Invalid Children's Aid Association. But your article of May 24 was so full of understanding of the almoner's point of view, that I trust these few lines may not give offence. My only object in writing is to secure mutual comprehension.

